# Presentation, management and outcome of aorto-esophageal fistula in young patients: two case-reports and literature review

**DOI:** 10.1093/jscr/rjab213

**Published:** 2021-06-29

**Authors:** Hassan Al-Thani, Bianca M Wahlen, Ayman El-Menyar, Ahmed Hussein, Ahmed Sadek, Amr Fares, Mohamed Musbah Saih, Abdulwahid Almulla

**Affiliations:** Trauma and Vascular Surgery Section, Department of Surgery, Hamad General Hospital (HGH), Doha, Qatar; Anaesthesiology Department, HGH, Doha, Qatar; Clinical Research, Trauma and Vascular Surgery Section, HGH, Doha, Qatar; Clinical Medicine, Weill Cornell Medical College, Doha, Qatar; Vascular Surgery Section, Department of Surgery, HGH, Doha, Qatar; Vascular Surgery Section, Department of Surgery, HGH, Doha, Qatar; Clinical Medicine, Weill Cornell Medical College, Doha, Qatar; Vascular Surgery Section, Department of Surgery, HGH, Doha, Qatar; Vascular Surgery Section, Department of Surgery, HGH, Doha, Qatar; Cardiothoracic Surgery, Heart Hospital, Hamad Medical Corporation, Doha, Qatar

**Keywords:** aortoenteric, aorto-esophageal, fistula, trauma, gastrectomy, endovascular repair, surgery

## Abstract

Aorto-esophageal fistula (AEF) is a rare serious surgical event. The first case developed hematemesis 2 weeks post-sleeve gastrectomy. A covered esophageal stent was placed endoscopically. The esophageal stent implantation was followed by massive bleeding due to an AEF. A thoracic endovascular aortic repair (TEVAR) was performed. Aggravated by infection of the aortic stent, another massive bleed occurred after 1 year. The final procedure was resection of the descending aorta with reconstruction using a bovine pericardial patch. The second case presented with hematemesis post-motor vehicle accident. AEF was confirmed by aortogram and treated by TEVAR followed by fully covered esophageal stent. The patient declined definite surgery. In conclusion, initial endovascular approach is useful as a bridge procedure. Once the patient hemodynamics are stabilized, a definitive surgical repair is required. The post-repair infection and life-long antibiotics could be overcome by using bovine pericardial grafts.

## INTRODUCTION

The incidence of aortoenteric fistulas including the aorto-esophageal fistula (AEF) is low; however, the mortality rate is high. The most common cause of AEF is thoracic aneurysm [1]. A revealing review by Hollander and Quick showed that the cause in 50% of cases was originally aortic disease, followed by foreign bodies and esophageal malignancy in 20% of cases. With less than 5%, secondary AEF has been reported in patients with aortic and esophageal surgery [2].

However, once a patient presents with a massive upper gastrointestinal (GI) hemorrhage, temporary measures such as the use of Sengstaken–Blakemore tube in order to apply direct pressure on the fistula from the esophageal side are useful [3]. Alternatively, if immediately available, embolization by an experienced interventional radiologist might be lifesaving.

While awaiting the final intervention, circulatory support such as inotropic use, volume replacement as well as correction of electrolyte and coagulation abnormalities should be the primary goal. Equally important is the administration of broad-spectrum antibiotics in order to prevent sepsis following an invasion of the esophageal flora into the mediastinum or aorta.

Current literature suggests various approaches to treat an AEF. One option is the conventional surgical repair. However, this approach is associated with high morbidity and mortality [4].

Another option for AEF treatment is the endovascular repair (TEVAR). The first successful endovascular repair of AEF was reported by Deshpande [5].

Nevertheless, there is an ongoing discussion whether the TEVAR in such cases can be the final solution or should be only used temporarily because of the potential risk of implanting a stent in an already infected area in addition to the risk of mechanical damage to the anatomical adjacent area through continuous arterial pulsation in the stented zone. Herein, we present two cases with AEF due to different causes in young patients with long-term follow-up.

## Case 1

**Figure 1:**
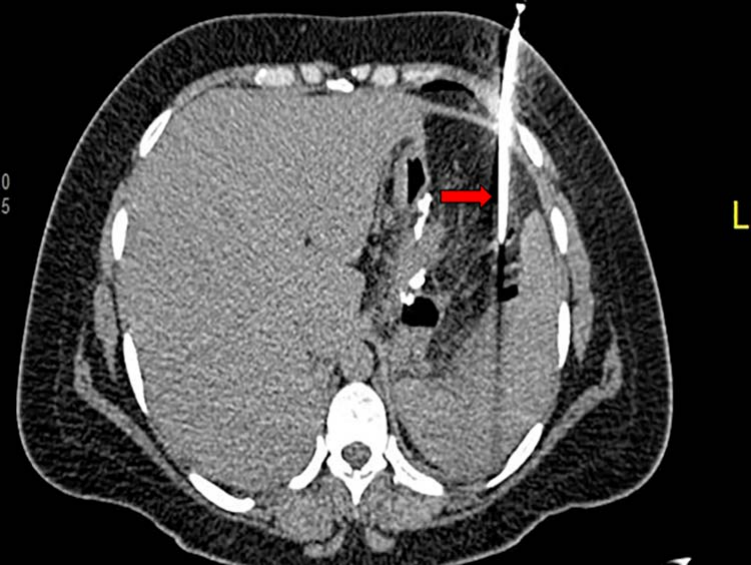
CT guided drain of intra-abdominal collection.

**Figure 2:**
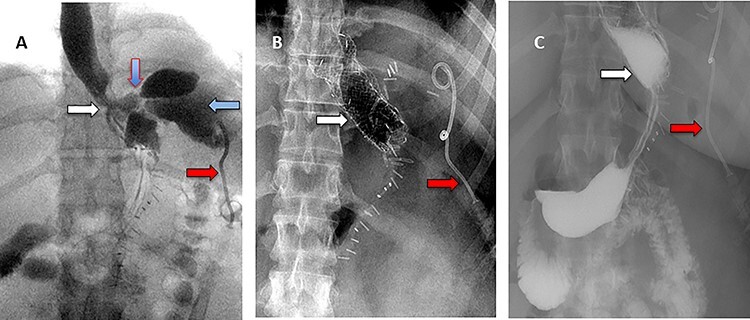
(**A**) Oral contrast medium showing communication between the lower esophagus and the site of collection drained by percutaneous pigtail catheter. (**B**) Distal esophageal stent was placed to cover the site of fistula. (**C**) No contrast extravasation.

**Figure 3:**
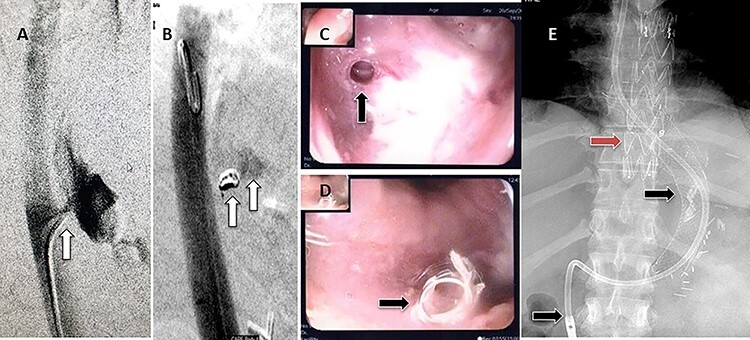
(**A**) Aortography showed active contrast extravasation to the esophagus trough the fistula. (**B**) Coli embolization of the AEF with contrast beyond the coil. (**C**) Esophagogastroduodenoscopy revealed the site of the fistula and the coil embolization in the stomach. (**D**) An esophageal stent was placed extending to the stomach and the aortic stent in place. Feeding tube in place.

**Figure 4:**
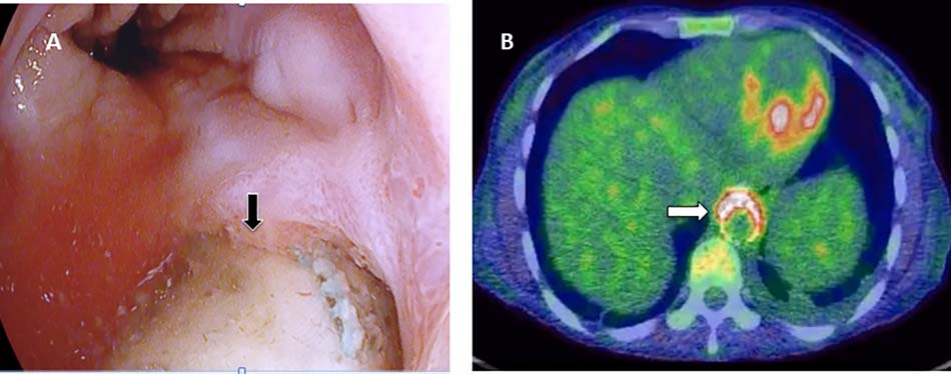
Esophagogastroduodenoscopy revealed thoracic endovascular aortic stent can be seen in the distal esophagus. (**B**) PET scan showing increase FDG activity around the distal aortic stent.

**Figure 5:**
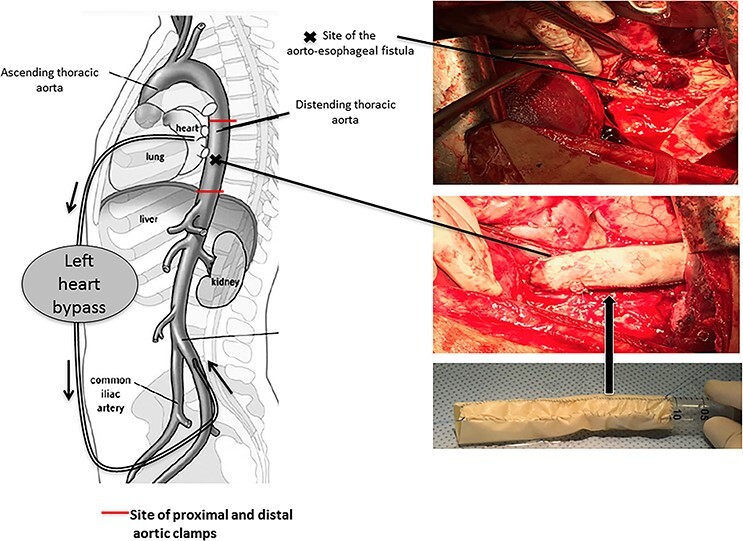
Pericardial bovine patch converted to a graft replacing the descent thoracic aorta at the site of fistula.

A 36-year-old obese female patient underwent a sleeve gastrectomy abroad. Eleven days after surgery, she presented with an abdominal pain. Abdominal computer tomography (CT) revealed several fluid-air collections (3 abscesses) around the upper greater gastric curve (3.5 × 3.2 cm), lesser sac (7 × 3.3 cm) as well as anterior to the spleen (7 × 3.3 cm). By that time, there was also left pleural effusion and atelectasis of the left lower lung lobe. On the next day, CT-guided aspiration was done (Fig. 1) and a pigtail catheter was inserted to drain the abscesses. On the following day, water-soluble oral contrast displayed an active contrast leak along the proximal sleeve gastrectomy (Fig. 2). On the same day, covered esophageal stent was placed endoscopically. The patient was discharged home and was scheduled for stent removal after 2 weeks. However, after 2 days, the patient presented again with an abdominal pain. The plain *x*-ray showed slippage of the stent distally. Upper GI endoscopy was done, and the esophageal stent was removed. One day later, the patient condition deteriorated with a massive upper GI bleeding. A CT angiography showed no extravasation and the source of bleeding was not identified during endoscopy due to massive bleeding. An immediately performed angiogram revealed an AEF (Fig. 3). The interventional radiologist achieved transient cessation of the bleeding through embolization of the fistula with interlock coils (Fig. 3). An aortogram showed continuous extravasation of contrast through the fistula, and therefore, endovascular intervention was performed with implantation of 22 mm × 112 mm aortic stent (TEVAR using Valiant covered stent - Medtronic company, USA). One week after TEVAR, another long esophageal stent was positioned (from lower esophagus to the stomach). Two weeks later, a barium swallow proved no evidence of contrast leak. Then, the esophageal stent was removed, and the patient was discharged home. There was a plan for definitive reconstruction procedure, including removal of the endovascular stent and use of reconstructed pericardial bovine graft, however, the patient declined. About 10 months later, she presented with another attack of massive hematemesis, with a drop of hemoglobin to 8 g/dl and a blood pressure of 66/44 mmHg requiring a rapid sequence induction and infusion of packed red blood cells. An immediate abdominal CT showed evidence of peri-stent infection with no contrast extravasation. Upper GI endoscopy showed an evidence of an ischemic ulcer above the Z-line with granulation tissue at the ulcer edge and erosion of the endovascular stent into the lower esophagus. The patient had a positron emission tomography (PET) scan and diagnosed with mediastinitis due to aortic stent infection and septic shock (Fig. 4). Surgical intervention was offered but the patient declined. Treatment with broad spectrum antibiotics and blood transfusion were administrated as required. Around 5 weeks later, the patient developed massive upper GI bleeding requiring intubation and surgical intervention (distal esophagectomy with removal of the endovascular stent and resection and replacement of the AEF site with reconstructed tube using 14 × 9 cm bovine pericardial graft through left thoraco-abdominal incision utilizing left cardiopulmonary bypass and distal perfusion through left femoral artery (Fig. 5). Closure of the stomach with gastrostomy tube insertion for feeding had been performed. One and half year later, reconstruction surgery of the esophagus with colonic interposition was done. After 3-year follow-up, the patient was doing well with no complication.

## Case 2

**Figure 6:**
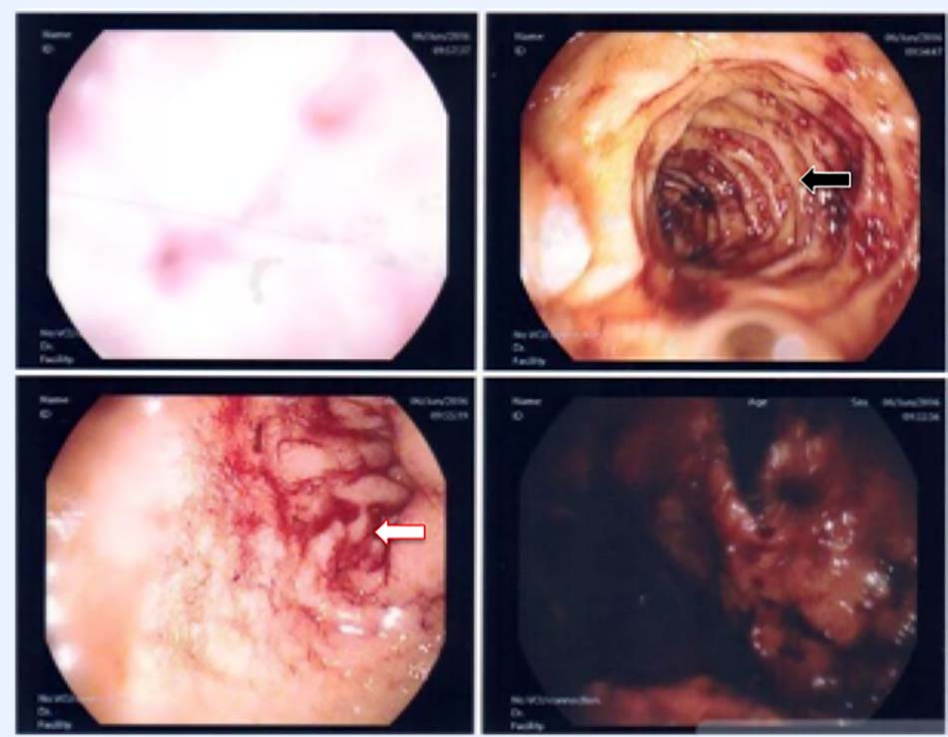
First upper endoscopy blood within the stomach and duodenum; the esophagus image was not clear and no active bleed can be seen.

**Figure 7:**
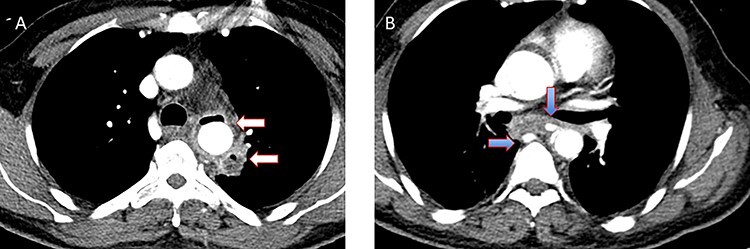
(**A**) CT study with contrast showing gas adjacent to the descending thoracic aorta with thickened soft tissue around the esophagus. (**B**) Vascular contrast within the esophagus and projecting outside the aorta forming ‘pseudoaneurysm’ or penetrating aortic ulcer.

**Figure 8:**
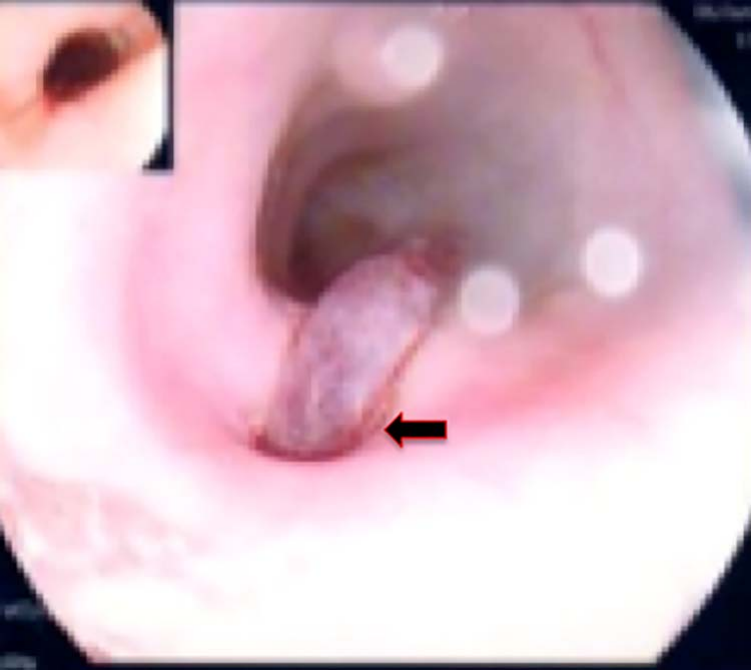
Second upper endoscopy fresh bleeding from the distal esophagus and after washing the blood an adherent clot extending from an ulcer going down into the stomach can be seen.

**Figure 9:**
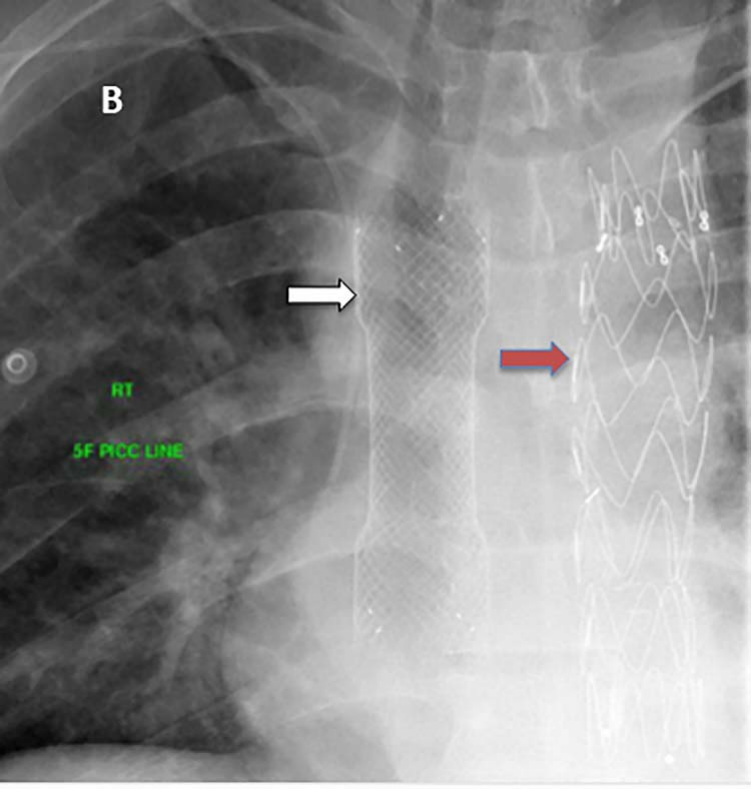
Esophageal stent (white arrow) and aortic stent (red arrow).

A 36-year-old male, involved in a minor motor vehicle accident while driving his car. He lost consciousness following a sudden onset of transient loss of vision which was not associated with palpitation or dizziness. His previous medical history revealed arterial hypertension and a corrected aortic coarctation with interposition graft at the age of 14. Pan CT scan was unremarkable except for thickening of the desending throacic aorta. His white blood count was very high (68 000 per cubic millimeter of blood). One day after the car accident, he developed nausea, hematemesis and melana. Upper GI endoscopy revealed blood in the stomach and distal esophafgus with no identifiable source of bleeding (Fig. 6). The patient developed massive hematemsis with hypotension requiring intubation and resuscitation. Another CT scan showed air pocket around distal aorta and contrast extravasation to the esophagus (Fig. 7). Another endoscopy revealed distal esophageal ulcer with clot extending toward the stomach (Fig. 8). AEF was suspected and then confirmed by aortogram. The fistula was treated by TEVAR (20 mm × 11.5 cm Valiant covered stent - Medtronic company, USA) followed by fully covered esophageal stent in the middle of the esophagus (Fig. 9).

The esophageal stent had been removed after 2 weeks. Six hours after stent removal, the patient showed again signs of active GI bleeding. Another upper GI endoscopy revealed superficial oozing from the site of the esophageal stent which was treated with cauterization and endoclip. Two month later, the patient was discharged with the recommendation for lifelong antibiotic therapy (Trimethoprim/sulfamethoxazole 800/160 mg) if no definitive reconstruction surgery done and Aspirin 100 mg. Three follow-up CT scans did not show any new pathology. Apart from one occasion of fever, the patient was doing well on regular outpatient clinic follow-up and he declined definitive surgery.

## DISCUSSION

An AEF defines the direct communication between the aorta and esophagus, with an annual incidence of 0.007 per million [6]. It is a rare but life-threatening surgical condition [7]. AEF diagnosis and treatment remain challenging. However, each treatment approach of AEF has its pros and cons. On the one hand, conventional open surgical repair may be associated with significant perioperative complications, such as bowel obstruction, mediastinal abscess and acute renal failure [8]. On the other hand, it is known that patients undergoing endovascular procedures such as stenting tend to have persistent infection-related complications (i.e. sepsis, abscess and endocarditis). Nevertheless, endovascular stent-graft used for bridging, until definitive surgical treatment performed, allows hemodynamic optimization in patients with blood loss and/or sepsis [9].

Overall, patients underwent open repair have longer survival and prolonged hospital length of stay when compared with their endovascular counterparts [8]. In general, the exact pathogenesis of AEF is often not well-defined, however few theories are presumed [2, 8–10]. Two major theories are competing when it comes to the development of AEFs, especially with previously inserted aortic stents. On the one side, the continuous arterial pulsation might be a possible cause for the development of such a fistula by creating a mechanical trauma in patients with pre-existing aortic stents. On the other side, there are repeatedly reported cases who developed sepsis after aortic grafts.

In those cases, an infection as the underlying cause with subsequent development of abscesses and finally destruction of the adjacent wall is assumed. This theory is underlined by the fact that, in up to 85% of patients who develop hemorrhage, the blood culture for enteric organisms is positive.

The second theory may be also underlined by the present first case where a seam insufficiency after sleeve gastrectomy seems to be the cause of infection and subsequent abscess, resulting in aortic erosion causing massive bleeding.

Without any surgical management, the survival rate of patients presenting with an aortoenteric fistula could be nil. In the present case, the patient underwent an immediate interventional radiology procedure with coiling and angioembolization.

A recent retrospective analysis [11] showed that additional coil embolization was a risk factor for infection of a stent. Literature [12] displays that its use can result in satisfactory short as well as long-term results; however, the importance of the underlying cause of the fistula should not be ignored. The initial use of TEVAR may result in further complications, such as leak, re-bleeding or infection after a not defined time frame [13].

In the first case, the final and life-saving treatment was surgical repair. This is underlined by literature stating that once complications occur, the final treatment is often an open surgical repair [14].

Takeno *et al.* [15] showed that the primary choice in the majority of AEF cases was TEVAR if the underlying cause was vascular. Esophagectomy in combination with TEVAR or graft replacement was advantageous in patients presented with an initial esophageal lesion and the combination of graft replacement with esophagectomy improved the prognosis, provided that long-term broad-spectrum antibiotics have been used.

The benefit of surgical treatment, once a graft or stent is infected, over conservative treatment is also underlined from Li *et al.*, in a recent meta-analysis [16]. In 2019, Carrel and colleagues confirmed this approach and emphasized that it is feasible for temporary bridging with a stent-graft only in selected subgroup of patients [17].

In general, on the long-term run, the avoidance of graft infection seems to be a major concern; therefore, life-long antibiotic therapy is recommended by some authors. However, this concern might be overcome by using a bovine graft. To our knowledge, the first case is unique in which aortoenteric fistula following sleeve gastrectomy was repaired using bovine pericardial graft.

## CONCLUSIONS

AEF is rare surgical event and its management is challenging. This report shows that an initial endovascular approach can be useful as a bridge until the patient is stabilized. Once the patient is hemodynamically stable, a definitive surgical repair is beneficial. The fear of infection and the negative impact of life-long antibiotics could be overcome after using a bovine pericardial graft.
